# Frequency‐Dependent Diffusion–Relaxation Distribution MRI: Scan–Rescan Reproducibility Ex Vivo and Caveats

**DOI:** 10.1002/nbm.70213

**Published:** 2025-12-25

**Authors:** Pak Shing Kenneth Or, Maxime Yon, Omar Narvaez, Eppu Manninen, Tarja Malm, Alejandra Sierra, Daniel Topgaard, Dan Benjamini

**Affiliations:** ^1^ Multiscale Imaging and Integrative Biophysics Unit National Institute on Aging, NIH Baltimore Maryland USA; ^2^ Department of Chemistry Lund University Lund Sweden; ^3^ Laboratoire Traitement du Signal et de l’Image Rennes University Rennes France; ^4^ A.I. Virtanen Institute for Molecular Sciences University of Eastern Finland Kuopio Finland

**Keywords:** diffusion MRI, frequency‐dependent diffusion, multidimensional MRI, oscillating gradients (OGSE), reproducibility, tensor valued diffusion encoding

## Abstract

Frequency‐dependent diffusion–relaxation distribution MRI provides information beyond the traditional voxel‐averaged metric that may better characterize the microstructural features of biological tissue. Frequency‐dependent multidimensional (ωMD) MRI reproducibility has been established in clinical settings, but has yet to be thoroughly evaluated under preclinical conditions, where superior hardware and modulated gradient waveforms enhance its performance. In this study, we investigate the reproducibility of ωMD‐MRI using a micro‐imaging system to investigate ex vivo mouse brains. Notably, the estimated signal fractions of intra‐voxel spectral components in the ωMD‐MRI distribution, corresponding to white and gray matter, along with the frequency‐dependent parameters, demonstrated high reproducibility. We identified bias between scan and rescan in some of the metrics, which we attribute to the time gap between repeated scans pointing to a long‐time progressive fixation effect. We compare our results with in vivo results from clinical scanners and show the reproducibility of diffusion frequency‐dependent metrics to benefit from the improved gradient hardware on our preclinical setup. Our results inform future micro‐imaging ex vivo studies of the reproducibility of ωMD‐MRI metrics and their dependence on fixation time.

## Graphical abstract

Frequency‐dependent multidimensional (ωMD) diffusion‐relaxation MRI captures microstructural information beyond conventional voxel‐averaged metrics. Here, we evaluate ωMD‐MRI reproducibility in ex vivo mouse brains using a high‐performance micro‐imaging system. Signal fractions associated with white and grey matter and frequency‐dependent parameters showed high reproducibility. Observed scan‐rescan biases likely reflect progressive fixation effects. Compared with clinical in vivo studies, reproducibility benefits from improved preclinical gradient hardware, informing future ex vivo ωMD‐MRI studies.

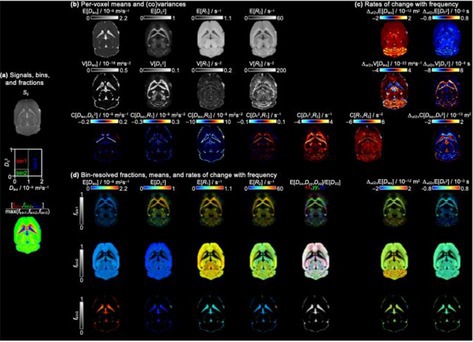

**Abbreviations**
$\tau_{E}$
: echo time
$\tau_{R}$
: recovery time
ω
: diffusion frequency
b
: diffusion encoding tensor
$b_{∆}$
: encoding anisotropy
$b\left(\omega \right)$
: diffusion encoding spectrum
CCC: Lin’s concordance correlation coefficient
CSF: cerebrospinal fluid
${\mathrm{CV}}_{\mathrm{ws}}$
: within‐subject coefficient of variation
D
: diffusion tensor
$D_{∆}\left(\omega \right)$
: frequency‐dependent normalized diffusion anisotropy
$D_{\mathrm{iso}}\left(\omega \right)$
: frequency‐dependent isotropic diffusivity
DTD: diffusion tensor distribution
EPI: echo planar imaging
FLASH: fast low‐angle shot
GM: gray matter
LOA: limits of agreement
MD: multidimensional diffusion
OGSE: oscillating gradient spin echo
$R_{1}$
: longitudinal relaxation rate
$R_{2}$
: transverse relaxation rate
ROI: region of interest
SNR: signal‐to‐noise ratio
WM: white matter

## 1 Introduction

In medical magnetic resonance imaging (MRI), tissue microstructure imaging is the characterization of tissue at the micrometer scale, which is typically two‐to‐three orders of magnitude finer than in vivo clinical MRI resolution [1]. The feat is challenging because of the complexity of tissue, which can consist of multiple, interacting or non‐interacting spin populations [2, 3] whose behaviors have not been fully characterized for different tissue types. This can be addressed through acquisition schemes that combine various diffusion encoding magnitudes and shapes [4], quantitative relaxometry [5], and tissue‐specific modeling [6, 7, 8]. Across different areas of nuclear magnetic resonance (NMR) and MRI applications, it has been shown that acquisition schemes that correlate two or more MR parameters can yield information that would otherwise remain inaccessible if the parameters were measured independently [9, 10, 11, 12]. A multidimensional MR measurement also enables the accounting of inherent biases that combining one‐dimensional measurements would have produced had they been acquired independently [5]. For example, signal fractions from different spin populations obtained by inversion using any model that does not account for relaxation may be misestimated in edema‐related conditions [7]. The wealth of information obtainable from diffusion–relaxation correlation studies has been demonstrated in detecting diffuse axonal injury [13], spinal cord injury [14, 15], neuroinflammation [16], placenta dysfunction [17], breast [18], and brain cancer [19], thus providing this modality great potential for patient diagnosis.

Another MRI modality that has been a topic of interest is diffusion spectrum encoding [20]. It is a method used to characterize the time dependence of diffusion behavior, which arises when water molecules diffuse in the presence of barriers that restrict their motion, as is likely the case in complex biological tissue. By varying the diffusion encoding waveform modulation or duration, the probed spectra of diffusion times or diffusion frequencies is also varied to control the amount of interaction between the water molecules and the barriers. Information about the barriers can then be extracted from the time dependence, adding an extra dimension of tissue characterization. Its utility has been demonstrated in understanding the mechanism of acute ischemic stroke [21], understanding epidermoid cysts [22], grading of intra‐axial brain tumors [23], and more.

The interpretation of a measurement can either be done through fitting to a biophysical model with assumptions specific to the measured tissue, or a general approach that makes fewer assumptions. While the biophysical modelling approach provides great interpretability to the results, incorrect model assumptions will lead to mischaracterization and misinterpretation [7, 24, 25, 26]. A general non‐parametric approach does not suffer from this pitfall, but is computationally expensive and prohibits direct use of a forward signal model for various purposes such as protocol optimization [27]. Nevertheless, for applications to samples that are as complex and heterogeneous as, for example, different types of tissue across the human brain, the cost of a non‐parametric approach can be a small price to pay to mitigate the risk of inaccurate model assumptions.

Frequency‐dependent multidimensional diffusion–relaxation correlation MRI (ωMD‐MRI) [28] is a technique that integrates several above‐mentioned MRI approaches into a single framework. Tensor‐valued diffusion encoding [29] is combined with diffusion spectrum encoding to probe the distribution of frequency‐dependent diffusion tensors [30], which are further correlated with relaxation measurements [28], while using a general parameter estimation method (Monte Carlo inversion [31, 32]) to untangle the multiple dimensions. As an all‐in‐one approach, we expect the technique to be able to characterize a wide range of tissue types and be useful in many research scenarios.

An efficient and sparse in vivo ωMD‐MRI clinical acquisition protocol that provides whole brain coverage at 2‐mm isotropic resolution was recently introduced [33]. The reliability and repeatability of the clinical ωMD‐MRI framework has been established, showing it to be comparable with conventional diffusion MRI reproducibility [34]. Human studies are essential for advancing the translation and application of ωMD‐MRI in clinical practice but have inherent limitations compared to preclinical animal models. Preclinical neuroimaging offers distinct advantages, including controlled experimental conditions, the ability to use complementary invasive techniques, access to genetic and disease models, and superior imaging hardware. These benefits are particularly valuable for studying the mechanisms underlying brain function and pathology. Recently, the application of ωMD‐MRI on a preclinical setup has been demonstrated on rats ex vivo [28] and in vivo [28, 35]. Before applying this technique to systematic comparative studies, our aim in the current study is to establish the reproducibility of a preclinical ωMD‐MRI framework by characterizing ex vivo mouse brains to assess intra‐scanner scan–rescan reproducibility.

## 2 Methods

### 2.1 Samples

Eight female mice, four of which were wild type (WT) and four were 5xFAD (a transgenic model to study familial Alzheimer's disease [36]), were perfused at approximately 8 months old with 0.9% saline for 2 min at 5 mL/min followed by phosphate‐buffered 4% paraformaldehyde solution for 20 min at 5 mL/min. The brains were extracted, trimmed to fit in 10 mm NMR tubes, preserved in phosphate‐buffered 2% paraformaldehyde solution and stored at 5 °C. The animal procedures were approved by the Animal Ethics Committee of the Province Government of Southern Finland and carried out according to the guidelines set by the European Community Council Directives 2010/63/EEC.

### 2.2 ωMD‐MRI Protocol Design

Optimization of protocol design has always been and still is an open research question. Here, we followed recently suggested heuristic description of the protocol and its design process [35]. Our protocol was designed to sample the widest possible range of parameters, within the limitations of the hardware, signal‐to‐noise ratio (SNR) with maximum SNR ~ 300 and minimum SNR ~ 7, after denoising, and scan time. The minimum echo time, $\tau_{E}$, is limited by the waveform duration, which in turn is limited by the gradient strength for a specific b value and modulation order. Because relaxation and diffusion weighting are both reflected in the signal by attenuation, the combination of maximum $\tau_{E}$, maximum b value and minimum recovery time (time between saturation pulses after one acquisition and excitation pulse of next acquisition), $\tau_{R}$, is limited by SNR.

Within these limitations, the protocol was designed following the following heuristic guidelines:

- When one weighting ($R_{1}$, $R_{2}$ relaxation, or diffusion) is low, the other two parameters are varied extensively. However, volumes where all three weightings are high are also included to avoid introducing bias into parameter correlations.
- Across b values, vary the diffusion encoding direction and modulation order to achieve a wide range of diffusion directions and frequencies.
- Focus the measurements on parts of the multidimensional acquisition parameter space that maximize signal differences between brain tissue types.

### 2.3 MRI Acquisition

**FIGURE 1:**
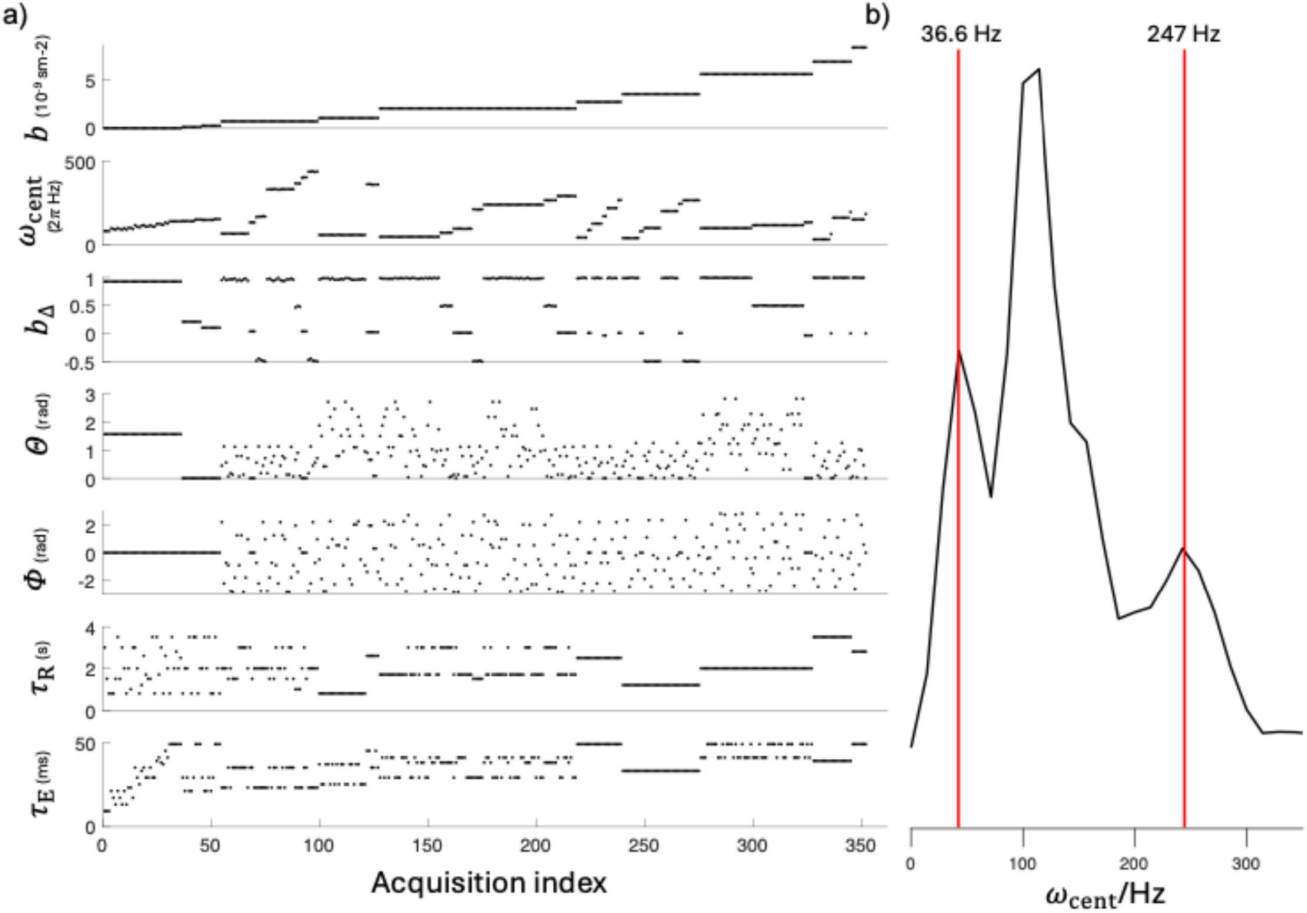
(a) ωMD‐MRI acquisition protocol of 352 images, acquired at different combinations of b values, centroid frequencies ($\omega_{\text{cent}}$) [37], b‐tensor shapes ($b_{∆}$), orientations of the diffusion encoding tensors (Θ,Φ), $\tau_{R}$, and $\tau_{E}$. (b) b Value‐weighted distribution of $\omega_{\text{cent}}$ of the protocol, effectively excluding low b value contributions. Red lines depict the 10th percentile and the 90th percentile.

All scans were acquired on a Bruker Avance‐III 11.7 T spectrometer (equipped with a MIC‐5 probe, 3 T/m maximum gradient amplitude) with the preclinical imaging software ParaVision 360. Each sample was scanned twice (2–7 months apart), using a ωMD‐MRI acquisition with echo planar imaging (EPI) readout, double sampling turned on, partial Fourier factor 6/8, 3D k‐space encoding, and 150‐μm isotropic resolution. Using the above protocol design heuristic guidelines, the acquisition of this study consisted of 352 volumes with $\tau_{R}$ ranging from 0.5 to 3.5 s, $\tau_{E}$ ranging between 10 and 50 ms, b values between 0 and 8.2 ms/mm^2^ limited by waveform durations 5 to 16 ms, and b‐tensor shapes, $b_{∆}$, of −0.5, 0, 0.5, and 1 (Figure 1). The protocol was repeated twice with AP and PA phase encoding directions to correct for EPI distortion, and the total acquisition time was ~26 h. As was the case in previous preclinical ωMD‐MRI studies [28, 35], strong gradients enabled the use of modulated diffusion gradients [37] that have high and narrow ranges in their encoding spectra while still achieving strong enough b values within acceptable $\tau_{E}$ s. Following the convention of Jiang et al. [37], waveforms with modulation orders 0 to 2 (related to the rotation angles of the diffusion gradients in the modulation scheme) were used in this study.

While both the initial scan and repeat scan (at a different date) of a sample were done at the same temperature setting (298 K), the flow rate of the variable‐temperature (vt) gas (which controls the sample temperature) was changed from 400 Lph for the first scans to 200 Lph for the second scans to reduce motion of the sample during acquisition. No effect on the data was observed.

Additional to the ωMD‐MRI acquisitions, a standard 3D FLASH scan at 50 μm resolution was acquired per sample to facilitate image registration, with $\tau_{E}$ = 10 ms, repetition time (time between excitation pulse of one acquisition and excitation pulse of the following acquisition) 0.7 s and flip angle = 30^o^ (scan time ~7 h).

### 2.4 Data Processing

#### 2.4.1 Preprocessing

After image reconstruction on ParaVision 360, denoising was done with MP‐PCA [38] with a patch size of 3 × 3 × 3, Rician bias correction applied directly on the magnitude images [39] using the noise variance estimate from MP‐PCA; Gibbs ringing removal was done on MRtrix3 [40], and EPI distortion correction was done using the TopUp function from FSL [41].

The FLASH acquisitions were not preprocessed.

#### 2.4.2 Parameter Estimation: ωMD‐MRI Inversion

As is done in previous studies [28, 34, 35], the estimation of ωMD‐MRI parameters [30] is performed using the multidimensional diffusion MRI toolbox for MATLAB [42], which employs a Monte Carlo inversion strategy [31, 32] to solve the inverse problem. The toolbox assumes the acquisition signal S in each voxel is represented by a sum of mono‐exponential components i

(1) $$\begin{array}{c} S\left(b\left(\omega \right),\tau_{R},\tau_{E}\right)=\sum_{i}w_{i}\;\exp\left(-\int_{\;-\infty }^{\infty }b\left(\omega \right):D_{i}\left(\omega \right)d\omega \right)\\ \left[1-\exp\left(-\tau_{R}R_{1,i}\right)\right]\exp\left(-\tau_{E}R_{2,i}\right) \end{array}$$

where $b\left(\omega \right)$ is the tensor‐valued encoding spectrum or dephasing power spectrum [43], $w_{i}$ the weight of each component, $D_{i}\left(\omega \right)$ the tensor‐valued diffusion spectra [44] in the laboratory frame of reference, and $R_{1,i}$ and $R_{2,i}$ the longitudinal and transverse relaxation rates of each component respectively. Each component is assumed to have axial symmetry in its principal axis system, which is rotated into the lab frame of reference through a rotational transformation $R\left(\theta_{i},\phi_{i}\right)$,

(2) $$D_{i}\left(\omega \right)=R\left(\theta_{i},\phi_{i}\right)\left(\begin{array}{ccc} D_{⊥,i}\left(\omega \right) & 0 & 0\\ 0 & D_{⊥,i}\left(\omega \right) & 0\\ 0 & 0 & D_{∥,i}\left(\omega \right) \end{array}\right)R^{-1}\left(\theta_{i},\phi_{i}\right)$$

The function forms of $D_{⊥,i}\left(\omega \right)$ and $D_{∥,i}\left(\omega \right)$ are parametrized with Lorentz distributions, characterized by the high frequency (i.e., restriction‐free diffusion) isotropic diffusivity $D_{0,i}$, and axial and radial transition frequencies ($\Gamma_{∥,i}$ and $\Gamma_{⊥,i}$) [30, 44] as

(3a) $$D_{∥,i}\left(\omega \right)=D_{0,i}-\frac{D_{0,i}-D_{∥,i}}{1+\omega^{2}/\Gamma_{∥,i}^{2}}$$

(3b) $$D_{⊥,i}\left(\omega \right)=D_{0,i}-\frac{D_{0,i}-D_{⊥,i}}{1+\omega^{2}/\Gamma_{⊥,i}^{2}}$$

For convenience of interpretation, the diffusivity parameters are expressed as isotropic diffusivity $D_{\mathrm{iso},i}\left(\omega \right)$ and normalized diffusion anisotropy $D_{∆,i}\left(\omega \right)$ by transforming $D_{⊥,i}\left(\omega \right)$ and $D_{∥,i}\left(\omega \right)$

(4a) $$D_{\mathrm{iso},i}\left(\omega \right)=\frac{D_{∥,i}\left(\omega \right)+2D_{⊥,i}\left(\omega \right)}{3}$$

(4b) $$D_{∆,i}\left(\omega \right)=\frac{D_{∥,i}\left(\omega \right)-D_{⊥,i}\left(\omega \right)}{3D_{\mathrm{iso},i}\left(\omega \right)}$$

Employing a bootstrapping strategy, an ensemble of solutions in the $D\left(\omega \right)-$ $R_{1}-R_{2}$ space is obtained for the same measurements. The ensemble of solutions collectively assesses the uncertainty in the solution landscape [31]. The solutions can be put together to form a continuous distribution of components [31, 32], which can further be quantified in terms of means (E[x]), variances ($V\left[x\right]$), and covariances ($C\left[x,y\right]$) at any specific ω. The analysis of the distributions is also done in bins in the $D_{\mathrm{iso}}$‐$D_{∆}^{2}$ space [45], which are defined as follows: bin 1: $D_{∆}^{2}$ > 0.25 and $D_{\mathrm{iso}}$ < 10^−9^ m^2^/s; bin 2: $D_{∆}^{2}$ < 0.25 and $D_{\mathrm{iso}}$ < 10^−9^ m^2^/s; bin 3: $D_{\mathrm{iso}}$ > 10^−9^ m^2^/s. Within the brain, these boundaries separate white matter (WM) from gray matter (GM), and the two from freely diffusing cerebrospinal fluid (CSF). Here, we focused on WM and GM regions that did not include CSF, and therefore *bin3* was not reported.

To evaluate how strongly $D_{\mathrm{iso}}\left(\omega \right)$ and $D_{∆}\left(\omega \right)$ depend on ω, their $E\left[x\right]$, $V\left[x\right]$ and $C\left[x,y\right]$ values at two different frequencies ($\omega_{\min}$ and $\omega_{\max}$) are evaluated to compute

(metrics) $${∆}_{\omega /2\pi }$$

(5) $${∆}_{\omega /2\pi }E\left[D_{\mathrm{iso}}\right]=\frac{E\left[D_{\mathrm{iso}}\left(\omega_{\max}\right)\right]-E\left[D_{\mathrm{iso}}\left(\omega_{\min}\right)\right]}{\frac{\omega_{\max}-\omega_{\min}}{2\pi }}$$

and similarly, for $V\left[x\right]$ and $C\left[x,y\right]$ metrics, and for $D_{∆}^{2}$.

Standard diffusion‐encoded color (DEC) maps [46] were made by finding the “median” fractional anisotropy (FA) and the “median” eigenvector. For each bootstrap solution, an FA and an eigenvector is defined by its mean diffusion tensor $\bar{D}\left(\omega \right)=\left\langle D\left(\omega \right)\right\rangle$, so a “median” can be defined over the bootstrap solutions. Notice that the mean diffusion tensor is a function of ω, so FA is also a function of ω.

In this approach, the only settings are the inversion limits of the solution space and inversion algorithm settings. For our data, the inversion limits used are 5 × 10^−12^ m^2^s^−1^ < $D_{0/⊥/∥}$ < 5 × 10^−9^, 0.1 s^−1^ < $\Gamma_{⊥/∥}$ < 10^5^ s^−1^, 0.1 s^−1^ < $R_{1}$ < 4 s^−1^, 4 s^−1^ < $R_{2}$ < 100 s^−1^. The other inversion algorithm settings are identical to that in Narvaez et al. [28]. Approximately, the inversion time for 130,000 voxels on a system with two 24‐core, 2.65 GHz CPUs was 40 h.

#### 2.4.3 Consolidation of Bootstrap Solutions and ROI‐Averaging

Two‐dimensional projections of the full $D\left(\omega \right)$‐$R_{1}$‐$R_{2}$ distributions were constructed to facilitate visualization and to allow investigation of certain regions of interest (ROIs). To obtain the distributions in each voxel, the bootstrap solutions first have to be consolidated into a single representative distribution. This was done using the following procedure: (1) grouping $D\left(\omega \right)$‐$R_{1}$‐$R_{2}$ components across different bootstrap solutions using *k*‐means clustering over [$D_{\mathrm{iso}}\left(\omega \right)$, $D_{\Delta }^{2}\left(\omega \right)$, θ, ϕ, $R_{1}$, $R_{2}$] as features (after normalization according to their respective maximal values) with the *L*1 distance metric. The *L*1 distance was chosen because compared to *L*2, it is more robust to outliers, better with sparse or high‐dimensional data, and computationally more efficient [47]. The number of clusters was defined as the median number of components observed across all bootstrap solutions. (2) Once grouped, each cluster is transformed into a single $D\left(\omega \right)$‐$R_{1}$‐$R_{2}$ component by taking the median of each parameter (e.g., $D_{\mathrm{iso}}$), and the median of the weights. This process then allowed the projection and mapping of the median weights of the discrete components from the consolidated distributions onto 64 × 64 meshes in the $D_{\mathrm{iso}}$‐$D_{\Delta }^{2}$, $D_{\mathrm{iso}}$‐$R_{1}$, and $D_{\mathrm{iso}}$‐$R_{2}$ planes. These voxel‐wise 2D projections were then summed over entire ROIs within each scan, normalized, and finally averaged across all scans to produce characteristic distributions.

#### 2.4.4 Image Registration and Region of Interest (ROI) Definition

**FIGURE 2:**
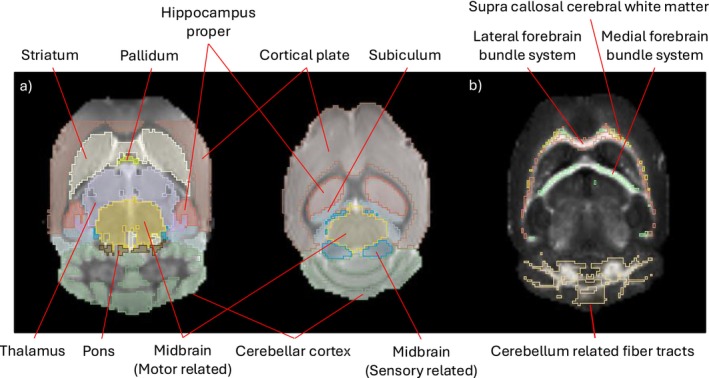
(a) Gray matter ROIs overlayed on top of a b0 structural image. (b) White matter fiber ROIs overlayed on top of a $E\left[D_{∆}^{2}\right]$ image.

By registering all scans to a mouse brain atlas, ROIs in native space were obtained for analysis, as seen in Figure 2 and detailed in Table 1. The atlas used is the Turone Mouse Brain Template and Atlas (NITRC—www.nitrc.org). The atlas provides a reference structural scan, which was used for registration and labels that delineate brain regions on the structural scan.

**TABLE 1:** Regions of interest (ROIs) used in the study.

ROI type	Regions of interest
Non‐fiber tracts	Cerebellar cortex, cortical plate, cortical subplate, hypothalamus, medulla, pallidum, pons, striatum, thalamus, behavioral state related, midbrain (motor related), midbrain (sensory related), hippocampus proper, dentate gyrus, entorhinal cortex, subiculum related areas
Fiber tracts	Cerebellum related fiber tracts, lateral forebrain bundle system, medial forebrain bundle system, supra callosal cerebral white matter

Advanced Normalization Tools [48] (ANTs version 2.5.1, http://stnava.github.io/ANTs/, accessed on 22 April 2024) was used for template creation and image registration. In brief, registration to the atlas reference consists of multiple steps, including creation of a mid‐way template between scan and rescan, and a study‐specific FLASH template. This was done utilizing three scans/maps, including a “b0” structural image for each scan, a DEC map for each scan, and a high‐resolution FLASH scan for each sample. The “b0” structural image was made by summing the images from six scans within the ωMD‐MRI protocol where b‐value = 0 such that a structural image representing that scan is obtained. Inclusion of the DEC maps enabled better registration of thin WM fibers. See Supplementary Information for a detailed description of the registration steps.

Regions of interest were grouped by brain region as defined by the Turone atlas (Table 1). The exceptions for this are the hippocampus proper, dentate gyrus, entorhinal cortex, and subiculum, which belong to the hippocampal formation. These ROIs do not include CSF voxels, and therefore *bin3* was not included in our analyses.

#### 2.4.5 Reproducibility Analysis

The reproducibility of each metric was assessed by comparing the ROI‐averaged value of the metric from the first scan of one sample to the repeat scan of the same sample. For binned metrics, the bin fraction‐weighted average is taken instead. Correlation plots were made for a qualitative assessment, and Lin's concordance correlation coefficient (CCC) [49] was computed for a quantitative assessment. CCC is a typical statistic used to measure reproducibility, taking not only Pearson's correlation into account but also the change in mean and variance between scan and rescan in its evaluation. The advantage CCC has over Pearson's correlation coefficient is that a high CCC can be achieved not only when the repeated measurements lie on a straight line but also the measurements have to be the same [50].

Bland–Altman plots were created to investigate the presence of bias by using MATLAB class “Bland‐Altman and Correlation Plot” [51]. Because the differences between scan and rescan were not Gaussian, the limits of agreement (LOAs) were estimated with the sample quantile estimator [52].

For completeness, CCC and Bland–Altman analyses were performed on the entire sample (*N* = 8), and separately on the WT and on the 5xFAD groups (*N* = 4 for both).

To be elaborated in the Discussion section, a systematic shift was observed in some of the metrics. The shifts were sample‐dependent and not related to the acquisition or processing. To factor out the bias, for each metric, Pearson's correlation coefficient was calculated for each sample to make boxplots for assessing “method‐specific” reproducibility.

To facilitate the comparison of reproducibility with other studies, the within‐subject coefficient of variation is computed. Following the definition in Manninen et al. [34], ${\mathrm{CV}}_{\mathrm{ws}}$ is estimated by

(6) $${\mathrm{CV}}_{\mathrm{ws}}\approx \frac{\sqrt{{\mathrm{MS}}_{\mathrm{ws}}}}{\left|\bar{\mu }\right|}$$

where ${\mathrm{MS}}_{\mathrm{ws}}$ is the within‐subject mean square difference and $\bar{\mu }$ is the mean over samples and scans.

## 3 Results

### 3.1 Regional Microstructural Characterization of the Mouse Brain

**FIGURE 3:**
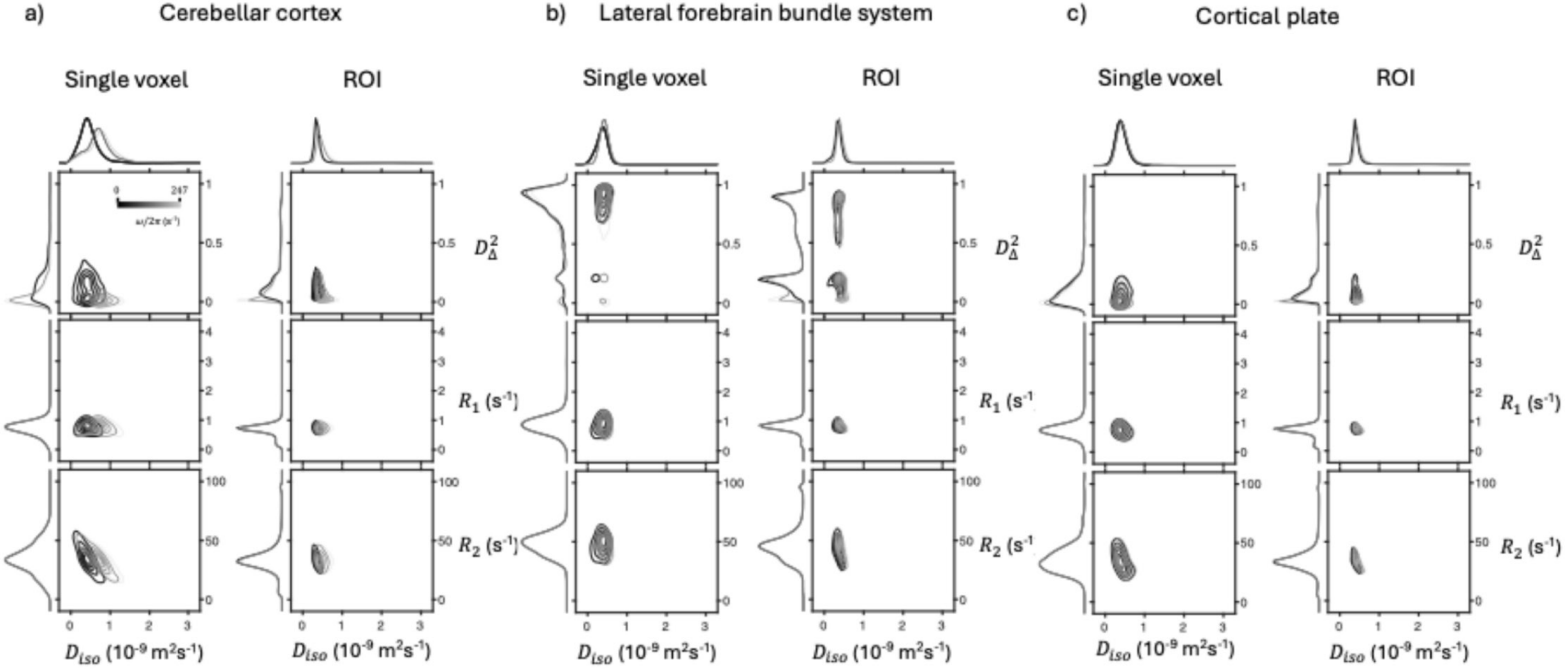
Projections of estimated $D\left(\omega \right)$‐$R_{1}$‐$R_{2}$ distributions in different regions of interest (ROI) and single voxels, from the WT samples. 2D projections on to the $D_{\mathrm{iso}}$‐$D_{∆}^{2}$, $D_{\mathrm{iso}}$‐$R_{1}$, and $D_{\mathrm{iso}}$‐$R_{2}$ dimensions from a single voxel (left column of each sub‐figure) and from an entire ROI (right column of each sub‐figure). On the edges of each plot are 1D projections to the corresponding dimension. Distributions at different diffusion frequencies ω/2π are plot with different shades of black. Here the distributions from the (a) cerebellar cortex, (b) lateral forebrain bundle system, and (c) cortical plate are shown.

Figure 3 shows projections of the bootstrap solution distribution in $D\left(\omega \right)$‐$R_{1}$‐$R_{2}$ space onto two and one dimension(s), obtained from single voxels and from entire ROIs in the WT group. Distributions of the entire ROIs are obtained according to the procedure described in the Methods section. Projections from three selected regions are presented to highlight their distinct characteristics.

The cerebellar cortex is shown for its strong $D_{\mathrm{iso}}$ and $D_{∆}^{2}$ frequency dependencies (i.e., ${∆}_{\omega /2\pi }E\left[D_{\mathrm{iso}}\right]$ and ${∆}_{\omega /2\pi }E\left[D_{∆}^{2}\right]$). This can be seen in the $D_{\mathrm{iso}}$‐$D_{∆}^{2}$ projection panels most easily in the single voxel distribution (Figure 3a, left column). The $D_{∆}^{2}$ changes from a wide distribution between 0 and 0.4 at the lowest frequency, to a singular peak near 0 at the highest frequency. Similarly, $D_{\mathrm{iso}}$ splits from a singular peak at the lowest frequency to two at the highest frequency. The lateral forebrain bundle system is shown as an example of highly anisotropic microstructure, illustrated in the single voxel $D_{\text{is}o}$‐$D_{∆}^{2}$ panel, the distribution is centered near the maximum value of 1 (Figure 3b, left column). The cortical plate was shown as an example of gray matter, which is characterized by low $D_{\mathrm{iso}}$ and $D_{∆}^{2}$, and a moderate response to diffusion encoding frequency. Two fractions, separable by $D_{∆}^{2}$, are detected, which merge at higher frequencies.

On the ROI‐averaged projections (Figure 3, right columns), we see a narrowing of peaks on the 1D projections which reflects the typical improvement of SNR by taking a larger sample size. However, ROI‐averaged results are also affected by partial volume effects from voxels at the boundaries of the regions, which possibly result in extra, distinct fractions that are not seen in the single‐voxel results. This is most severe in the lateral forebrain bundle system ROI, due to its long and narrow nature, creating a low $D_{∆}^{2}$ fraction that is approximately the amplitude of the high $D_{∆}^{2}$ fraction.

**FIGURE 4:**
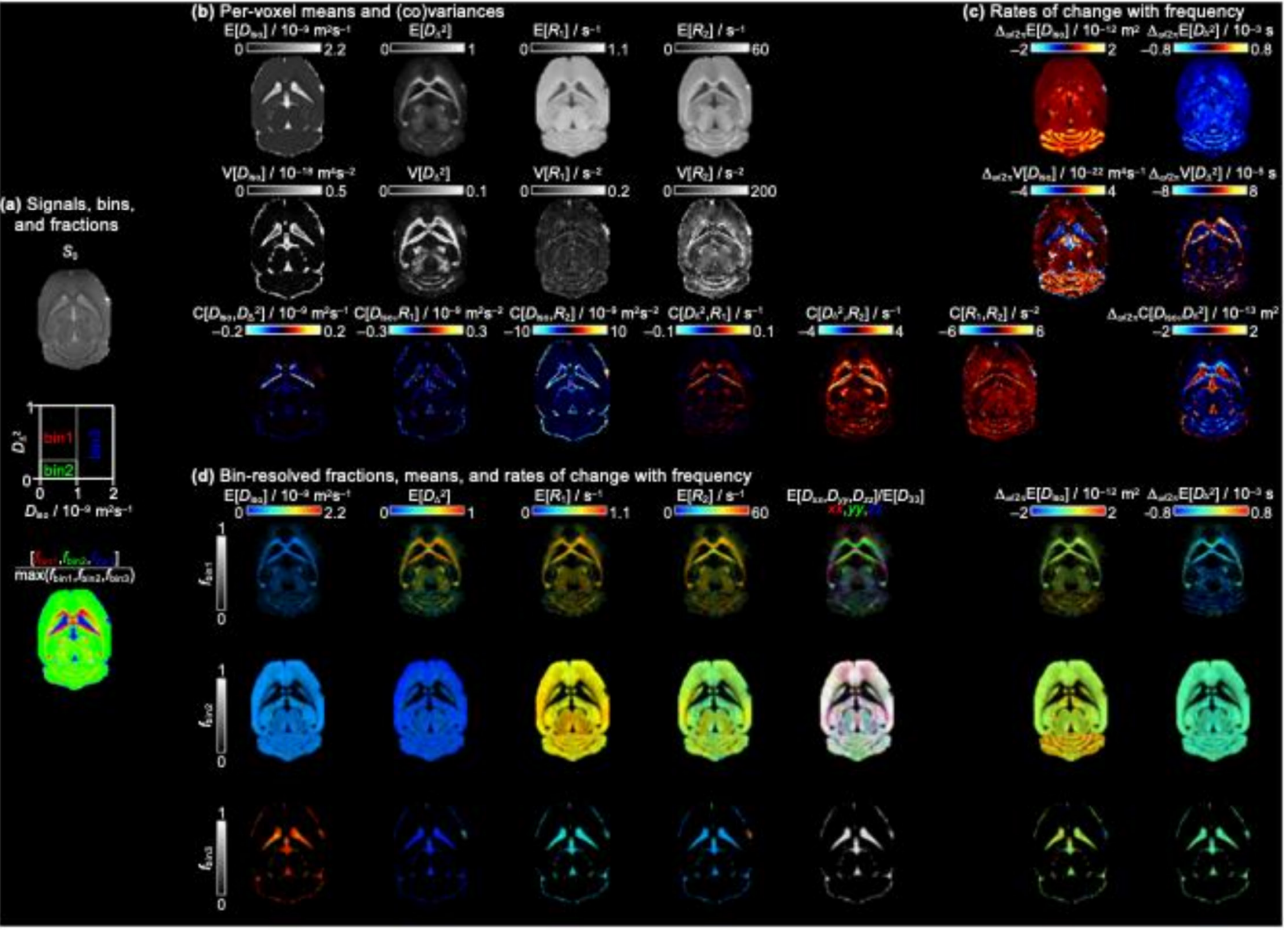
Parameter maps in a transvers slice from a WT mouse brain. (a) Signal amplitude map estimated by the solution, binning scheme and dominant bin composition of each voxel. (b) Per‐voxel means, variances, and covariances. (c) Diffusion encoding frequency dependence of $D_{\mathrm{iso}}$ and $D_{∆}^{2}$, as described by Equation (3). (d) Bin‐resolved, per‐voxel means of MRI metrics.

The voxelwise distributions can be summarized by their means ($E\left[x\right]$), variances ($V\left[x\right]$), and covariances ($C\left[x,y\right]$) over the entire solution space. This straightforward dimensionality reduction allows for visualization of the essential relaxation–diffusion characteristics in a representative slice from one of the ex vivo WT mouse brains, as shown in Figure 4.

### 3.2 Test–Retest Reproducibility

**FIGURE 5:**
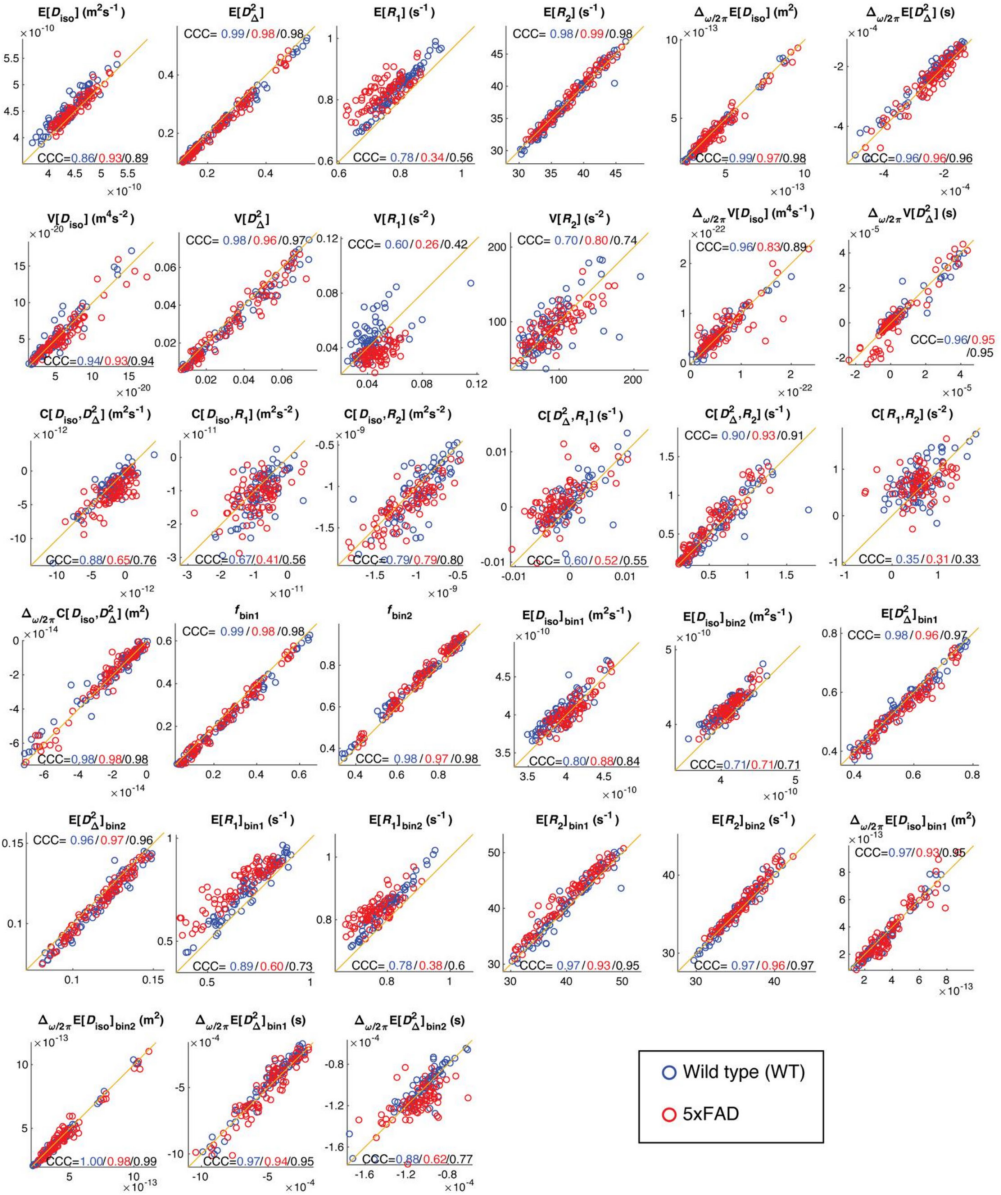
Correlation plots of ωMD‐MRI metrics and their CCCs for per‐voxel metrics and bin‐resolved per‐voxel metrics. Each point on the plot is the ROI‐averaged value of the metric from a sample from one of the ROIs, from the first scan for its *x*‐coordinate and rescan for its *y*‐coordinate. Data from WT and 5xFAD mice are color‐coded as blue and red, respectively.

Figure 5 presents the correlation plots and CCCs for each parameter. Each point represents the ROI‐averaged value from either WT (blue) or 5xFAD (red) mice. CCC values are shown for all samples combined (black), as well as separately for WT (blue) and 5xFAD (red) groups. Overall, we observed greater repeatability in the healthy group compared to the diseased mice. Following established recommendations [53], we rated the reproducibility of each parameter according to their CCCs, summarized in Table 2.

**TABLE 2:** ωMD‐MRI metrics rated by reproducibility based on CCC (in the WT group).

Strength‐of‐agreement (CCC)	Metrics
Excellent (> 0.899)	$E\left[D_{\Delta }^{2}\right]$, $E\left[R_{2}\right]$, ${∆}_{\omega /2\pi }E\left[D_{\mathrm{iso}}\right]$, ${∆}_{\omega /2\pi }E\left[D_{∆}^{2}\right]$, $V\left[D_{\mathrm{iso}}\right]$, $V\left[D_{\Delta }^{2}\right]$, ${∆}_{\omega /2\pi }V\left[D_{∆}^{2}\right]$, ${∆}_{\omega /2\pi }V\left[D_{\mathrm{iso}}\right]$, $C\left[D_{∆}^{2},R_{2}\right],$ ${∆}_{\omega /2\pi }C\left[D_{\mathrm{iso}},D_{∆}^{2}\right]$, $f_{\mathrm{bin}1}$, $f_{\mathrm{bin}2}$, $E{\left[D_{\Delta }^{2}\right]}_{\mathrm{bin}1},E{\left[D_{\Delta }^{2}\right]}_{\mathrm{bin}2},E{\left[R_{2}\right]}_{\mathrm{bin}1}$, $E{\left[R_{2}\right]}_{\mathrm{bin}2}$, ${∆}_{\omega /2\pi }E{\left[D_{\mathrm{iso}}\right]}_{\mathrm{bin}1}$, ${∆}_{\omega /2\pi }E{\left[D_{\mathrm{iso}}\right]}_{\mathrm{bin}2}$, ${∆}_{\omega /2\pi }E{\left[D_{∆}^{2}\right]}_{\mathrm{bin}1}$
Strong (0.800–0.899)	$E\left[D_{\mathrm{iso}}\right]$, $C\left[D_{\mathrm{iso}},D_{∆}^{2}\right]$, $E{\left[D_{\mathrm{iso}}\right]}_{\mathrm{bin}1}$, $E{\left[R_{1}\right]}_{\mathrm{bin}1}$,${∆}_{\omega /2\pi }{\left[D_{∆}^{2}\right]}_{\mathrm{bin}2}$
Moderate (0.600–0.799)	$E\left[R_{1}\right],V\left[R_{1}\right],V\left[R_{2}\right]$, $C\left[D_{\mathrm{iso}},R_{1}\right],C\left[D_{\mathrm{iso}},R_{2}\right]$, , $E{\left[D_{\mathrm{iso}}\right]}_{\mathrm{bin}2}$, $E{\left[R_{1}\right]}_{\mathrm{bin}2}$
Fair (0.300–0.599)	$C\left[D_{∆}^{2},R_{1}\right]$, $C\left[R_{1},R_{2}\right]$

**FIGURE 6:**
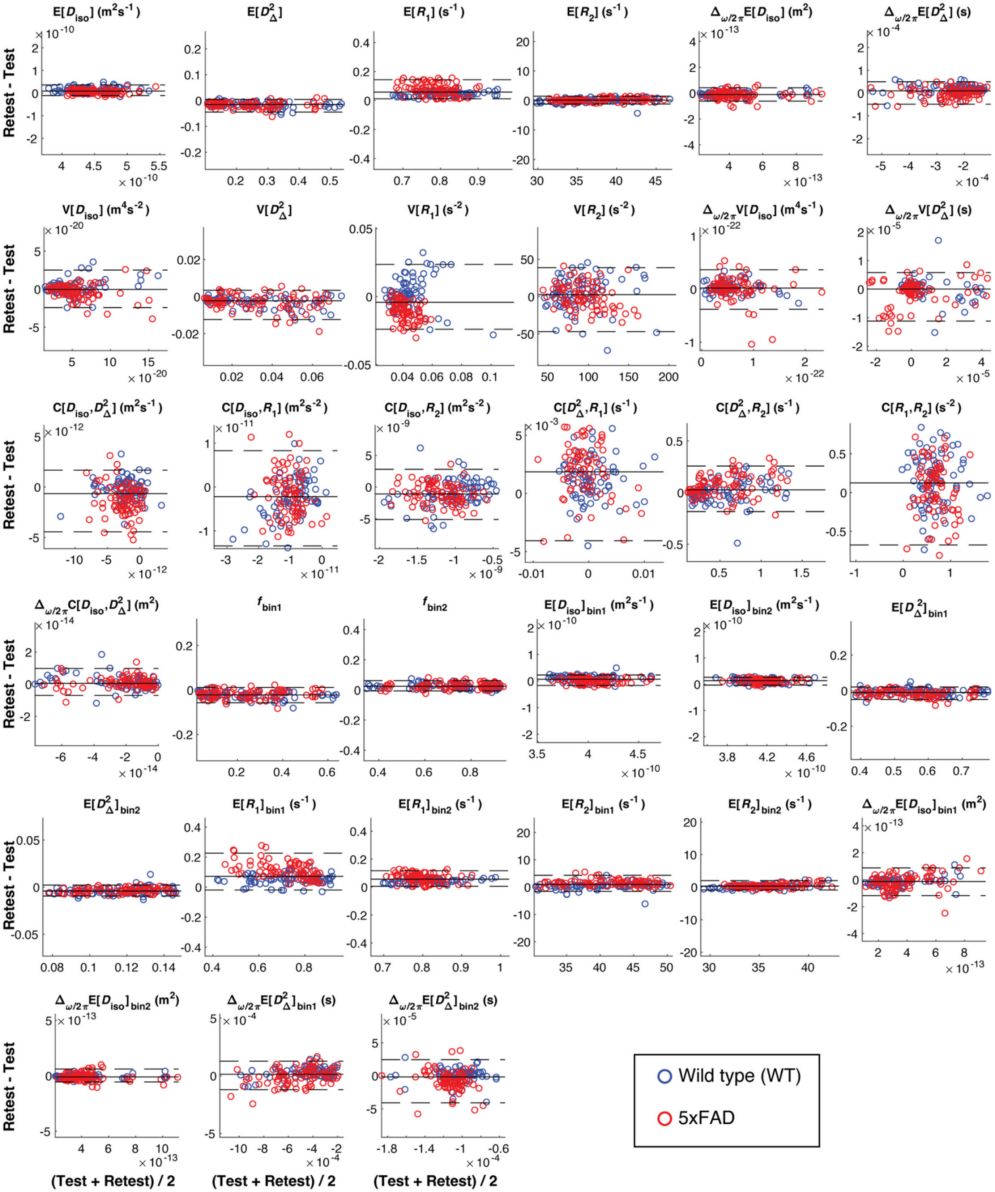
Bland–Altman plots for per‐voxel metrics and bin‐resolved per‐voxel metrics. On each plot, there is a solid line that depicts the mean “rescan–scan” difference, and two dotted lines depicting the limits of agreement (LOA). *y*‐Axis of plots are scaled to 80% of the maximum average measurement to facilitate comparison, so for poorly performing metrics one or both LOA lines can be out of bounds. Data from WT and 5xFAD mice are color‐coded as blue and red, respectively.

Figure 6 shows the Bland–Altman plots, quantifying the difference between scan and rescan against the average of the measurements. Same as in Figure 5, data from WT and 5xFAD mice are color‐coded as blue and red, respectively. Bias in Bland–Altman plots is indicated when the LOA do not include zero, which is the case for $E\left[R_{1}\right]$ and $E{\left[R_{1}\right]}_{\mathrm{bin}2}$.

**FIGURE 7:**
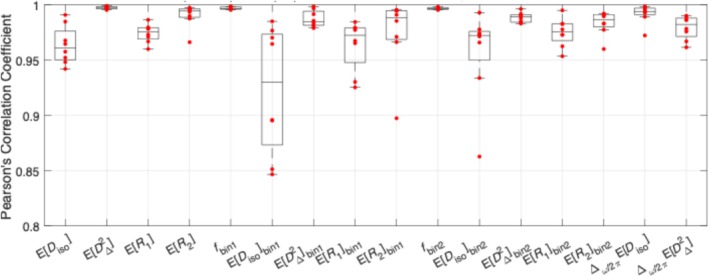
Box plots for per‐sample Pearson correlation coefficients of selected metrics.

In the correlation plot of $E\left[R_{1}\right]$, we observe significant bias between scan and rescan. We further noticed that the deviation of the data points from the unity line increases with increasing time between scans, while maintaining a similar slope of one. We identified the source of this bias to be long‐term interaction between the sample and the preserving paraformaldehyde solution (detailed in the Discussion, Section 4.5). Thus, to isolate the bias related to sample condition from “method‐specific” reproducibility, we calculate Pearson's correlation coefficient for each sample separately, and show the results as box plots (Figure 7). We focused on metrics with detectable or suspected bias, describing them by median Pearson's correlation coefficients and IQRs: $E\left[R_{1}\right]$ (0.994; 0.989–0.996), $E{\left[R_{1}\right]}_{\mathrm{bin}1}$ (0.972; 0.948–0.979), $E{\left[R_{1}\right]}_{\mathrm{bin}2}$ (0.975; 0.968–0.983), $E\left[D_{\mathrm{iso}}\right]$ (0.961; 0.950–0.976), and $E{\left[D_{\mathrm{iso}}\right]}_{\mathrm{bin}2}$ (0.972; 0.950–0.976). All show median correlations above 0.95, indicating excellent agreement.

For $E{\left[D_{\mathrm{iso}}\right]}_{\mathrm{bin}1}$, the four best‐performing samples show noticeably higher reproducibility than the other four (Figure 7), suggesting that reduced reproducibility is driven by these specific samples rather than by a systematic bias such as time in paraformaldehyde. This separation does not appear to be related to sample type (WT vs. AD), fixation time at either scan, or the interval between scans, and could be explained by sub‐optimal registration of WM regions.

## 4 Discussion

Diffusion–relaxation correlation MD‐MRI enables detailed tissue microstructure characterization by mapping multidimensional distributions of MRI contrasts. This approach enhances sensitivity and specificity by isolating distinct diffusion–relaxation spectral ranges, reducing signal averaging effects. In this study, we investigated the reproducibility of ωMD‐MRI metrics using ex vivo, preserved mice brains. This study systematically evaluated regional multicomponent diffusion–relaxation properties across white matter tracts, cortical gray matter, and subcortical gray matter, revealing distinct microstructural differences. Additionally, a test–retest analysis demonstrated that the reproducibility of ωMD‐MRI parameters is comparable to alternative metrics, supporting its reliability for pre‐clinical brain imaging.

### 4.1 Regional Microstructural Features

As seen in previous ωMD‐MRI studies [28, 34, 35], the 2D projections characterize different tissue types well. In WM voxels, we find high $D_{∆}^{2}$ values, and in GM voxels, we find low $D_{∆}^{2}$ values. More notably, the analysis reveals the presence of multiple distinguishable components within a single voxel, where the variations between these compartments are more subtle than the pronounced differences observed between anisotropic WM and isotropic GM. For example, in the single voxel and ROI‐averaged $D_{\mathrm{iso}}$‐$D_{∆}^{2}$ projection of the cortical plate in Figure 3c, it can be seen that the $D_{∆}^{2}$ dimension separates the distribution into two. Recently, Yon et al. [35] also demonstrated a detection of two compartments in GM in in vivo rat brain scans with ωMD‐MRI 2D projections, separated by $D_{\mathrm{iso}}$. The reproducibility of these smaller compartments should be investigated in a future study.

### 4.2 Reproducibility Comparison With Clinical ωMD‐MRI

**FIGURE 8:**
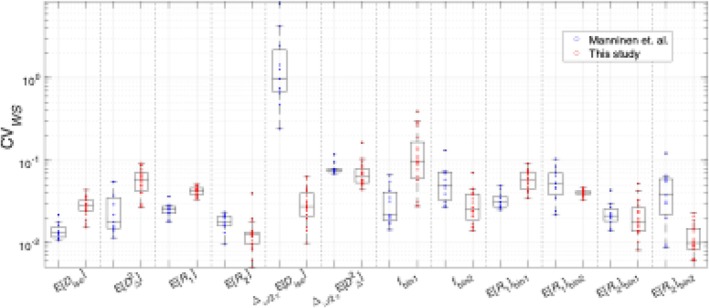
Box plots for ROI‐specific ${\mathrm{CV}}_{\mathrm{ws}}$ for selected ωMD‐MRI metrics, retrieved from Manninen et al. and calculated for the data in this study. Outlier criterion is value > 1.5x interquartile range from 25th or 75th percentile (box edge).

The only other scan–rescan study for ωMD‐MRI was done recently by Manninen et al. [34] on healthy human participants on a 3‐T clinical scanner. Reproducibility was quantified using the intraclass correlation coefficient (ICC), and variability was quantified using within‐subject coefficient of variation (${\mathrm{CV}}_{\mathrm{ws}}$). Due to the small sample size that further separates into two groups (WT and 5xFAD transgenic mice) of this study, ICC cannot be reliably estimated in the same manner. Using an online calculator [54] recommended in the literature [55], for an expected ICC of 0.9, the setup of this study would result in a 95% confidence interval of [0.65, 1.15], which includes three out of the four ICC classes recommended by Koo and Li [56]. We therefore only estimated ${\mathrm{CV}}_{\mathrm{ws}}$ for our data, separately for the two groups of mouse brains, to facilitate direct comparison with the in vivo ωMD‐MRI results. A side‐by‐side comparison of ${\mathrm{CV}}_{\mathrm{ws}}$ values from Manninen et al. and from the WT mice in the current study is shown in Figure 8.

In general, metrics that performed well in Manninen et al. also performed well in this study. The most striking differences are the much better performance of ${∆}_{\omega /2\pi }E\left[D_{\mathrm{iso}}\right]$ and the poorer performance of $f_{\mathrm{bin}1}$ in our study. We attribute the better ${∆}_{\omega /2\pi }E\left[D_{\mathrm{iso}}\right]$ performance to the larger diffusion encoding frequency range in the acquisition protocol of this study, the $\omega_{\text{cent}}$ of which in Manninen et al. was 6.6–21 Hz and in this study is 36.6–247 Hz. The poorer performance of $f_{\mathrm{bin}1}$ is likely due to the lower WM content in the mouse brain compared to the human brain, resulting in lower $f_{\mathrm{bin}1}$ values in this study. In Manninen et al., all ROIs have $f_{\mathrm{bin}1}$> 0.3. In our study, for ROIs with ${\mathrm{CV}}_{\mathrm{ws}}$> 0.1 in the $f_{\mathrm{bin}1}$ parameter, $f_{\mathrm{bin}1}$ means are smaller than 0.2. As ${\mathrm{CV}}_{\mathrm{ws}}$ is a ratio between measurement standard deviation and measurement mean, a smaller denominator naturally gives a larger coefficient of variation.

Although ICCs from Manninen et al. cannot be directly compared with our CCCs, it is notable that their in vivo study found the frequency‐sensitive parameters (${∆}_{\omega /2\pi }E\left[D_{\mathrm{iso}}\right]$ and ${∆}_{\omega /2\pi }E\left[D_{∆}^{2}\right]$) to be the least reproducible, whereas in our study, they rank among the most reproducible. We again attribute the discrepancy to the larger diffusion encoding frequency range in the acquisition protocol of this study.

When analyzing the reproducibility of the WT and 5xFAD groups separately, we observed a slight advantage of the former (Figures 5 and 6). This result likely reflects the greater heterogeneity introduced by amyloid pathology, gliosis, and neurodegeneration. These tissue alterations modify relaxation and diffusion properties, reduce SNR, and increase sensitivity to artifacts, all of which contribute to lower concordance across repeated scans.

### 4.3 Reproducibility of Variance and Covariance Metrics

Not investigated in Manninen et al., variance and covariance ($V\left[x\right]$ and $C\left[x,y\right]$) metrics can also be extracted from the bootstrap inversion solution. In theory, the variances of the solution component distribution describe the diversity in microenvironments in the voxel, but in practice, they also describe the uncertainty in the solution space due to the presence of noise [31]. The high reproducibility of the diffusion variance and covariance parameters in this study (e.g., $V\left[D_{\mathrm{iso}}\right]$, ${∆}_{\omega /2\pi }V\left[D_{∆}^{2}\right]$, and ${∆}_{\omega /2\pi }C\left[D_{\mathrm{iso}},D_{∆}^{2}\right]$) suggests that the MRI acquisition protocol sufficiently samples the diffusion‐weighting space and leads to the also high reproducibility of bin fraction estimation. Conversely, the lower reproducibility of $V\left[R_{1}\right]$ and $V\left[R_{2}\right]$ suggests that the acquisition protocol might be improved by incorporating more variation in $\tau_{R}$ and $\tau_{E}$ to stabilize the solution for the relaxation rates.

Apart from noise sensitivity, partial volume also negatively affects the reproducibility of $V\left[x\right]$ and $C\left[x,y\right]$ metrics, more than $E\left[x\right]$ metrics. The exact placement of a voxel on a boundary between different tissue types (e.g., CSF/paraformaldehyde vs. GM) affects the ratio of mixing. While the dependence of $E\left[x\right]$ metrics on mixing ratio is monotonous determined by the two means of the two voxel types, the dependence of $V\left[x\right]$ and $C\left[x,y\right]$ metrics is non‐linear (parabolic for the case of $V\left[x\right]$). For the sake of illustration, consider the $R_{2}$ of a voxel with a partial mix of paraformaldehyde and GM (respectively, means: 10 s ^− 1^ and 30 s^−1^, variances: 10 s^−2^ and 50 s^−2^). While the $E\left[R_{2}\right]$ of the voxel lies between the two means, $V\left[R_{2}\right]$ reaches a maximum of ~140 s ^− 2^ at ~0.5 mixing ratio. The expected absolute difference of $E\left[R_{2}\right]$ and $V\left[R_{2}\right]$ between two scans can then be computed, giving ~7 s^−1^ and ~40 s^−2^ respectively.

### 4.4 Systematic Bias of ωMD‐MRI Metrics

For any reproducibility study, systematic bias is not usually expected. Here, systematic bias was found for $E\left[R_{1}\right]$ and $E{\left[R_{1}\right]}_{\mathrm{bin}2}$, and suspect borderline bias in $E\left[D_{\mathrm{iso}}\right]$, $E\left[D_{∆}^{2}\right]$, $f_{\mathrm{bin}1}$, $E{\left[R_{1}\right]}_{\mathrm{bin}1}$, $f_{\mathrm{bin}2}$, and $E{\left[D_{\mathrm{iso}}\right]}_{\mathrm{bin}2}$. It may seem counterintuitive that some metrics show both high reproducibility and suspected bias. We argue this occurs because their variability is low enough that even small biases, relative to the measurement scale, become detectable.

Two possible sources of bias are the change in the flow rate of the vt gas between the first and the second repeat scans (changed from 400 to 200 Lph), and changes in sample condition due to long‐time progressive fixation. A follow‐up experiment scanning one sample twice back‐to‐back at the two different flow rates was done to disentangle the two factors (see Section 2 in the Supplementary Information and Supplementary Figure S1). Bias was not seen in the new results, leading us to conclude that the cause is fixation‐driven chemical alteration. Although the effects of fixation on sample condition time scales relevant to our study (> 4 months after fixation, 3–7 months between scan and rescan) have not been investigated in the literature, relaxation rates have been shown to progressively increase at shorter times [57], and diffusion rates also have been shown to increase [58]. A clear dependence of $E\left[R_{1}\right]$ on fixation time is seen across all ROIs (Supplementary Figure S1), indicating that the scan–rescan interval influences $R_{1}$ values. Thus, the reduced reliability of $R_{1}$ reflects true physical changes rather than issues with acquisition or processing. Of note, these results demonstrate that many diffusion and transverse relaxation parameters, which did not show bias, are very insensitive to long‐term fixation effects.

### 4.5 Reproducibility Comparison With Other MRI Studies

Coelho et al. investigated the reproducibility of 2D diffusion‐$R_{2}$ correlation metrics in the human brain in vivo using a clinical scanner [8]. Like ωMD‐MRI, they used a variable TE and tensor valued encoding imaging sequence. Unlike ωMD‐MRI's non‐parametric estimation method, they model their signal with the “standard model” of white matter [59], which in brief is a three‐compartment signal model with diffusion and transverse relaxation contributions, and estimate the model parameters with machine learning inversion. They presented voxelwise CCC values for the model parameters calculated from two repeated scans from a single volunteer. In Table 3, we categorize the standard model parameters into four categories, and do the same for ωMD‐MRI parameters for a side‐by‐side comparison with Coelho et al. We make two observations: first, the ωMD‐MRI parameters perform better, which can be attributed to the ROI‐wise evaluation of CCCs, a lengthier and thus more thorough acquisition protocol, and better gradient hardware. Second, similar to our results, their anisotropy parameter outperforms their diffusivity parameters.

**TABLE 3:** Comparison of CCC reproducibility findings from the current study and two other studies.

	Coelho et al. [8]		Zhong et al. [60]		Current study	
	Metric	CCC (voxelwise)	Metric	CCC	Metric	CCC
Bin fraction	f $f_{w}$	0.95 0.75	NODDI_ICVF NODDI_ECVF NODDI_ISOVF	0.86 0.88 0.89	$f_{\mathrm{bin}1}$ $f_{\mathrm{bin}2}$	0.99 0.98
Anisotropy	$p_{2}$	0.97	DTI_FA DKI_FA	0.94 0.93	$E\left[D_{∆}^{2}\right]$ $E{\left[D_{∆}^{2}\right]}_{\mathrm{bin}1}$ $E{\left[D_{∆}^{2}\right]}_{\mathrm{bin}2}$	0.99 0.98 0.96
Diffusivity	$D_{a}$ $D_{e}^{∥}$ $D_{e}^{⊥}$	0.88 0.90 0.72	DTI_MD DKI_MD	0.85 0.86	$E\left[D_{\mathrm{iso}}\right]$ $E{\left[D_{\mathrm{iso}}\right]}_{\mathrm{bin}1}$ $E{\left[D_{\mathrm{iso}}\right]}_{\mathrm{bin}2}$	0.86 0.80 0.71
Relaxation	$T_{2,a}$ $T_{2,e}$	0.79 0.77	n/a	n/a	$E\left[R_{1}\right]$ $E\left[R_{2}\right]$	0.78 0.98

Another study by Zhong et al. [60] investigated scan–rescan reproducibility of diffusion MRI metrics estimated from data acquired with a conventional multi‐shell diffusion MRI protocol using four popular diffusion models (DTI [61], DKI [62], MAP [63], and NODDI [6]). Relevant parameters from the four models are also shown in Table 3 for comparison with our results. Again, metrics from our study performed better overall, and anisotropy metrics performed better than diffusivity metrics.

### 4.6 Inadequate Noise Estimation Reduces Diffusion Frequency Sensitivity

Noise suppression is essential in modern MRI to improve repeatability and mitigate the Rician noise floor without losing sample information. In this study, we applied MP‐PCA [38] for noise estimation and suppression, followed by Rician bias correction on the magnitude images using the MP‐PCA‐derived noise estimate [39].

However, in our multidimensional acquisition, the assumption of redundancy is not fully valid because the encoding space is high‐dimensional and sparsely sampled for practical reasons. This sparse sampling introduces substantial spatial signal heterogeneity, which can compromise the accuracy of the MP‐PCA‐derived noise estimate and, consequently, influence the Rician bias correction. To minimize these effects, we chose a MP‐PCA patch size of 3 × 3 × 3 for this study, acknowledging it may have an over‐smoothing effect in our data. And indeed, the largest effect of the Rician bias correction was observed in the diffusion anisotropy frequency parameter, ${∆}_{\omega /2\pi }E\left[D_{∆}^{2}\right]$: without correction, cerebellar fiber tracts showed strong diffusion frequency sensitivity, whereas after correction, voxels with strong sensitivity adjacent to weak‐sensitivity voxels also became weak (Supplementary Figure S2), an effect not clearly reflected in the CCC value of the metric. The loss of diffusion frequency contrast could also be driven by a sample‐ or protocol‐specific effect.

A potential solution to avoid the reduction in diffusion frequency dynamic range and parameter sensitivity is to suppress noise in the complex data, where it is Gaussian and the noise floor is inherently removed. This was not tested here due to lack of access to complex data.

Nonetheless, Rician bias correction was applied in this study, as we observed an improvement in CCC for $E\left[D_{\mathrm{iso}}\right]$ (Supplementary Figure S3), which is likely the result of the reduction of an arbitrarily slow diffusion component from the suppression of the noise floor.

### 4.7 Limitations

The small sample size is a limitation that affected the analyses in this study. Aside from prohibiting the use of conventional reproducibility metrics such as ICCs and coefficients of variation, we also opted for a joint analysis of all ROIs instead of individual ROIs separately.

Because the focus was on brain microstructure, ROIs were deliberately chosen to exclude areas with significant CSF content. Also, $f_{\mathrm{bin}3}$ in non‐CSF voxels are general near 0. As a result, the amount of free water within the analyzed ROIs was minimal. In such regions, even small variations or estimation errors between test and retest sessions can result in large relative differences, leading to low repeatability metrics [34]. We therefore chose to exclude bin3 parameters from our analyses. However, this raises a broader question about how best to define distribution binning in multidimensional MRI: Should it rely on fixed “spectral” regions or be guided by data‐driven approaches? In this study, we adopted previously established fixed spectral regions [28], but other ωMD‐MRI studies have successfully implemented data‐driven binning strategies [13, 16, 64]. We believe this is a critical and evolving area of research that should be actively explored and optimized based on the specific biological question being addressed.

Despite quality assurance by inspection at every step of the image registration procedure, misregistration unavoidably reduces the reproducibility values calculated in this study. Because we investigate the reproducibility of ROI‐averages, the effect of misregistration for GM regions is expected to be small due to their low surface area‐to‐volume ratio. On the other hand, WM tract ROIs are generally long and thin. At the ωMD‐MRI resolution used in this study, mouse WM tracts contain portions that are 1 voxel thin, and thus are more susceptible to misregistration. Sub‐optimal registration in WM would explain the relatively heterogeneous performance of $E{\left[D_{\mathrm{iso}}\right]}_{\mathrm{bin}1}$ (Figure 7). For the same reason, WM tracts are also susceptible to partial volume effects, a significant portion of which contain a mix of GM and WM.

To minimize these effects, the simplest solution would be to increase the spatial resolution of the ωMD‐MRI acquisition. However, the resolution of EPI readout is limited by the sample's apparent transverse relaxation rate ($R_{2}^{*}$) due to its reliance on gradient echoes. Increasing the echo train number to sample a larger k‐space, thus increasing spatial resolution, is ineffective, as signal loss occurs at the train's end. Increasing the number of readout segments could be a workaround, but that would at least double the already lengthy scan time which was impractical for us.

Alternatively, readout methods that are not heavily limited by $R_{2}^{*}$ relaxation such as RARE [65] and GRASE [66] can be used. However, EPI has the advantage of achieving a shorter echo time, which is important for this study because of the high fast transverse relaxation of the samples.

In general, the reproducibility assessment of ωMD‐MRI in this study is not generalizable to all ωMD‐MRI studies. Under a different setup (e.g., different field strength, acquisition settings such as resolution and readout), the SNR of the acquisition will be different, which affects the precision of the inversion. We have also seen that the design of the preprocessing pipeline changes the inversion results, which will have an effect on reproducibility. Moreover, Monte Carlo inversion is unbiased in the high‐SNR limit, but its parameter estimates become increasingly variable and biased as noise rises. At lower SNR, fitted distributions spread around the ground truth, subtle components may be lost, and parameter coupling exacerbates cross‐talk, reducing reproducibility and accuracy. These effects have been demonstrated in simulations and experiments, highlighting the critical dependence of non‐linear parameter fitting on SNR [32].

## 5 Conclusion and Future Work

In this study, we investigated the reproducibility of ωMD‐MRI metrics on ex vivo mouse brain samples and showed metrics that are highly repeatable, and metrics that are sensitive to the conditions of the samples measured but otherwise also repeatable when bias is excluded. We offer plausible explanations to the changes in the conditions, which informs future ex vivo ωMD‐MRI studies.

Overall, diffusion–relaxation first and second order parameters (e.g., $E\left[D_{∆}^{2}\right]$ and $V\left[D_{\mathrm{iso}}\right]$), frequency‐dependent diffusion parameters, and some of the binned metrics (e.g., $f_{\mathrm{bin}2}$, $E{\left[R_{2}\right]}_{\mathrm{bin}2}$) performed similarly or better than the reproducibility of MRI metrics found in the literature.

One of the most significant findings here is the remarkable reproducibility of the frequency‐dependent parameters, ${∆}_{\omega /2\pi }E\left[D_{\mathrm{iso}}\right]$ and ${∆}_{\omega /2\pi }E\left[D_{∆}^{2}\right]$ (while acknowledging the sensitivity of ${∆}_{\omega /2\pi }E\left[D_{∆}^{2}\right]$ to the preprocessing pipeline). Because of their potential to measure microstructural features through restricted diffusion, methods that probe frequency‐dependence have been studied since the 1990s [67]. Yet, the reproducibility of these ωMD‐MRI metrics has not been demonstrated in a clinical setting. In fact, as discussed, they have been shown to be unreliable [34]. We hypothesize that the reason those metrics are repeatable here but not on human scanners is the smaller diffusion encoding frequency ranges achievable on typical human scanners (6.6–21 Hz vs. 36.6–247 Hz). These results thus motivate further optimization of diffusion‐encoding waveforms to maximize frequency, and improvement of magnetic gradient hardware [68] on clinical scanners to enable higher encoding frequencies.

To date, multiple versions of ωMD‐MRI protocols already exist on clinical scanners [19, 33] with the most recent rendition achieving full brain coverage at 2 mm isotropic resolution on a 3‐T Philips scanner at a scan time of ~20 min (unpublished). In translating our preclinical protocol to clinical settings, two key challenges must be addressed: the limited scan times that constrain the number of parameters that can be sampled, and hardware restrictions that narrow the range of diffusion‐encoding spectral frequencies. These factors will be critical considerations when seeking to reproduce preclinical findings in the clinic.

## Author Contributions

P.S.K.O.: investigation, formal analysis, visualization, writing – original draft preparation. M.Y.: methodology, investigation. O.N.: methodology. E.M.: methodology, software. T.M.: methodology. A.S.: methodology, resources. D.T.: conceptualization, resources, supervision, writing – review and editing. D.B.: conceptualization, project administration, funding acquisition, resources, supervision, visualization, writing – review and editing. All authors contributed to the final version of the manuscript.

## Funding

This study is supported by the National Institutes of Health; Stiftelsen för Strategisk Forskning, ITM17‐0267; Vetenskapsrådet, 2018‐03697, 2022‐04422_VR, 21073; and Research Council of Finland, 361370, 358944.

## Ethics Statement

The research was conducted according to the principles expressed in the Declaration of Helsinki. The animal study was reviewed and approved by the Animal Committee of the Provincial Government of Southern Finland.

## Conflicts of Interest

The authors declare no conflicts of interest.

## Supporting information


**Figure S1:** (a) Scan–rescan values of R1 across different samples. For each data point, the *x*‐coordinate is an ROI's R1 value evaluated at the first scan of a sample, and *y*‐coordinate is that evaluated at the repeat scan of the same sample. The data points are color coded by how far apart in time the scans are (as illustrated in the color bar on the right), where darker colors represent data points from which the time between scans is smaller, and lighter colors represent that with longer time in between scans. The group of solid‐color data points are from a pair of scans performed 1 day apart. As it can be seen, the darker the color, the close the data points are to the line of unity (yellow line). (b) ROI‐averaged R1 values from the same subject, scanned at 3 time points. Each line represents the R1 value of the same ROI evaluated from the 3 scans from different time points. A general increasing trend can be observed.
**Figure S2:** Δω/2πEDΔ2 map from a representative sample without (left) and with (right) Rician bias correction.
**Figure S3:** CCC values computed from results with and without Rician bias correction, both denoised with MP‐PCA patch size 3 × 3 × 3. Note the improvement in EDiso and EDisobin2 when bias corre.

## Data Availability

MATLAB source code for preprocessing and Monte‐Carlo data inversion is freely available at https://github.com/maximeYon/MMD. The acquisition sequence is available upon reasonable request depending on Paravision versions.
