# Supplementary material for: Frequency‐Dependent Diffusion–Relaxation Distribution MRI: Scan–Rescan Reproducibility Ex Vivo and Caveats
Source: NMR Biomed. 2025 Dec 25;39(2):e70213. doi: 10.1002/nbm.70213 (PMC12741536; doi:10.1002/nbm.70213)
Supplement: Supplementary file 1 — Figure S1: (a) Scan–rescan values of R1 across different samples. For each data point, the x‐coordinate is an ROI's R1 value evaluated at the first scan of a sample, and y‐coordinate is that evaluated at the repeat scan of the same sample. The data points are color coded by how far apart in time the scans are (as illustrated in the color bar on the right), where darker colors represent data points from which the time between scans is smaller, and lighter colors represent that with longer time in between scans. The group of solid‐color data points are from a pair of scans performed 1 day apart. As it can be seen, the darker the color, the close the data points are to the line of unity (yellow line). (b) ROI‐averaged R1 values from the same subject, scanned at 3 time points. Each line represents the R1 value of the same ROI evaluated from the 3 scans from different time points. A general increasing trend can be observed. Figure S2: Δω/2πEDΔ2 map from a representative sample without (left) and with (right) Rician bias correction. Figure S3: CCC values computed from results with and without Rician bias correction, both denoised with MP‐PCA patch size 3 × 3 × 3. Note the improvement in EDiso and EDisobin2 when bias corre. [file NBM-39-e70213-s001.pdf]

Supplementary Information: Frequency-dependent diffusion-relaxation distribution MRI: scan-rescan reproducibility ex vivo and caveats

Pak Shing Kenneth Or[1,2], Maxime Yon[2,3,4], Omar Narvaez[4], Eppu Manninen[1], Tarja Malm[4], Alejandra Sierra[4], Daniel Topgaard[2] and Dan Benjamini[1*]

[1]Multiscale Imaging and Integrative Biophysics Unit, National Institute on Aging, NIH, Baltimore, MD, USA
[2]Department of Chemistry, Lund University, Lund, Sweden
[3]Laboratoire Traitement du Signal et de l'Image, Rennes University, Rennes, France
[4]A.I. Virtanen Institute for Molecular Sciences, University of Eastern Finland, Kuopio, Finland

*Correspondence to:
Dan Benjamini, PhD
The National Institute on Aging
251 Bayview Blvd. Baltimore, MD 21224, USA
Email: dan.benjamini@nih.gov

Section 1: Detailed registration steps

Registration-related processing was done with the Advanced Normalization Tools (ANTs) toolbox[1] and MATLAB (Natick, Massachusetts: The MathWorks Inc.).

The first step of the registration is to combine the b_0 image from each of the two scans of each sample into an intermediate template. This is done with the “antsMultivariateTemplateConstruction2” command. It registers any number of similar volumes to each other and creates an averaged volume, or template in the terminology of ANTs. The processed μFA map was also supplemented to the template construction algorithm to ensure better alignment of the thin, fiber tracks. A b_0 template and a processed μFA template for each sample is obtained from this step.

The second step is to register the b_0 template of each sample to their respective FLASH scans. Because of the resolution difference, the FLASH scans were down-sampled to the resolution of the md-MRI scans with the *imresize* MATLAB function. The first part this registration is to make a processed μFA map for the FLASH scan. This was done by rigidly registering the processed μFA map of the first scan to the FLASH scan with the “antsRegistrationSyN” command. The second part is to register the B0 template to the FLASH with the additional input of the processed μFA template and the FLASH-registered processed μFA map. This is done with the “antsRegistration” command, which allows multimodal registration.

The third step is to make a FLASH template from the individual FLASH scans. We found that registering the FLASH scans to the reference first before template creation preserves small and thin structures better. Thus, first we register each FLASH scan to the atlas reference with “antsRegistrationSyN” with the “-t s” option that performs rigid, affine and then deformable registration in that order. The registered FLASH scans were then used to create a FLASH template with “antsMultivariateTemplateConstruction2”.

The final registration step is to register the FLASH template to the atlas reference, simply with the “antsRegistrationSyN” with the only the deformable registration option.

In one of the FLASH scans, susceptibility artefacts were found, and they translated into the FLASH template. The scan was thus excluded from template creation. To transform the FLASH from that scan to the FLASH template, the scan was registered to the template with “antsRegistration” with its processed μFA map and a white matter mask obtained from by transforming the white matter mask that comes with the atlas into the FLASH template space as additional input.

After the above registration steps, all scans are transformed to the atlas reference. The brain region labels are then translated from atlas space to native (scan) space with linear interpolation. Before this step, ROIs are stored as binary matrices; voxels

that are part of an ROI are notated 1 and those that are not, are notated 0. Interpolation transforms the matrices into a continuous distribution of values, so it is returned to binary form by applying a threshold of 0.8. This also serves as an ROI eroding step to reduce effects from misregistration.

Voxels that were predominantly composed of $bin3$ (i.e., high diffusivity) components, which in this study correspond to the fixing solution of the samples. Thus, voxels where $f_{\text{bin3}} > 0.5$ were removed from the ROIs.

Finally, the ROIs are manually adjusted by inspection. Using 3D Slicer[2], the ROIs were overlayed on top of the b0 and μFA maps, and any mismatch or susceptibility artefacts were removed from the final ROIs. The ROIs “cranial nerves” and “extrapyramidal fiber systems” were removed as part of the procedure.

Section 2: Long-term effect of fixing solution on R_1 of samples

As can be seen from Supplementary Figure 1b, a clear R1 vs fixation time dependency is observed in all ROIs. As such, the scan-rescan interval is affecting the R1, and therefore the apparent reliability of the R1 parameters is reduced. This apparent poor reliability of R1 is driven by actual changes in R1 and not by the acquisition or processing pipelines.

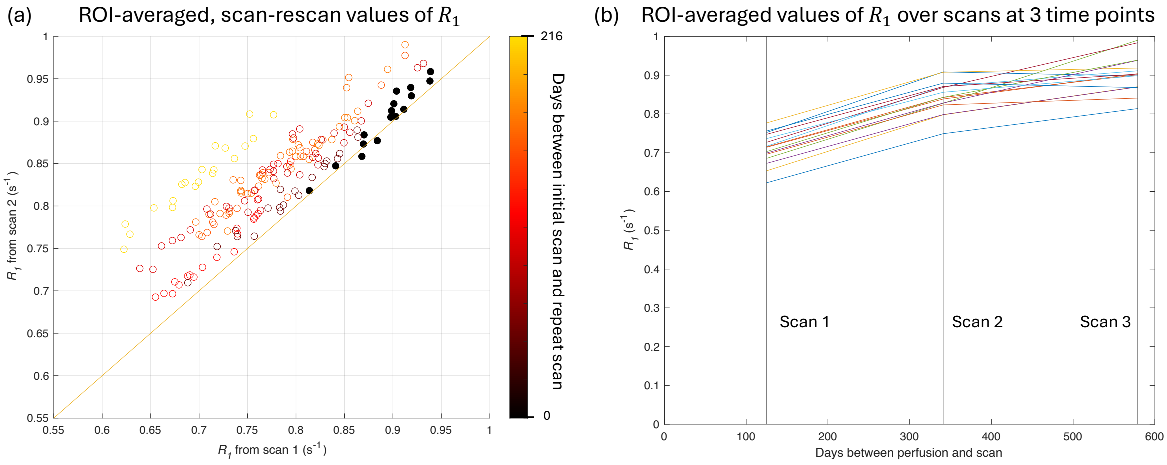

Supplementary Figure 1. (a) Scan-rescan values of R1 across different samples. For each data point, the x-coordinate is an ROI’s R1 value evaluated at the first scan of a sample, and y-coordinate is that evaluated at the repeat scan of the same sample. The data points are color coded by how far apart in time the scans are (as illustrated in the color bar on the right), where darker colors represent data points from which the time between scans is smaller, and lighter colors represent that with longer time in between scans. The group of solid-color data points are from a pair of scans performed 1 day apart. As it can be seen, the darker the color, the close the data points are to the line of unity (yellow line). (b) ROI-averaged R1 values from the same subject, scanned at 3 time points. Each line represents the R1 value of the same ROI evaluated from the 3 scans from different time points. A general increasing trend can be observed.

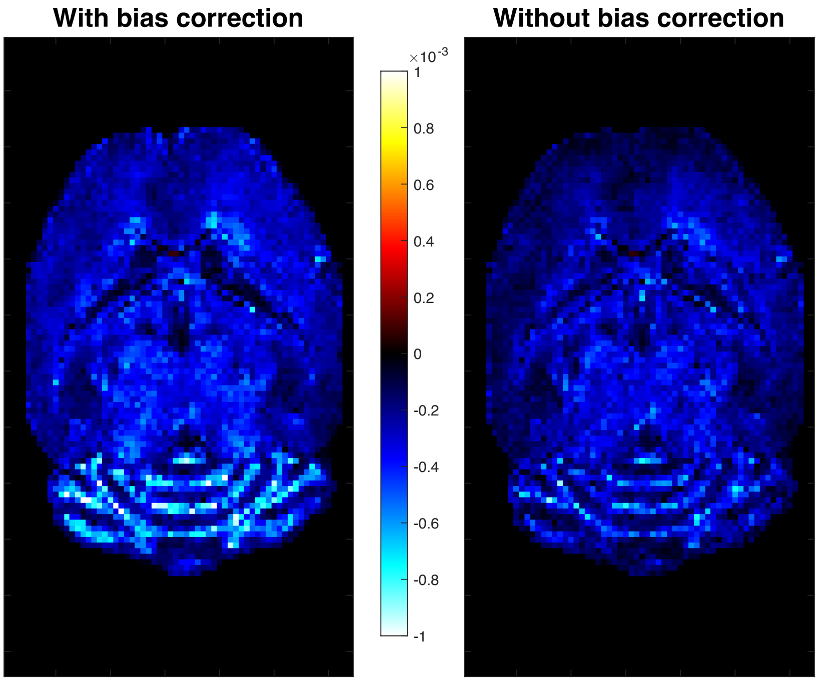

Supplementary Figure 2. $\Delta_{\omega/2\pi}E[D_{\Delta}^{2}]$ map from a representative sample without (left) and with (right) Rician bias correction.

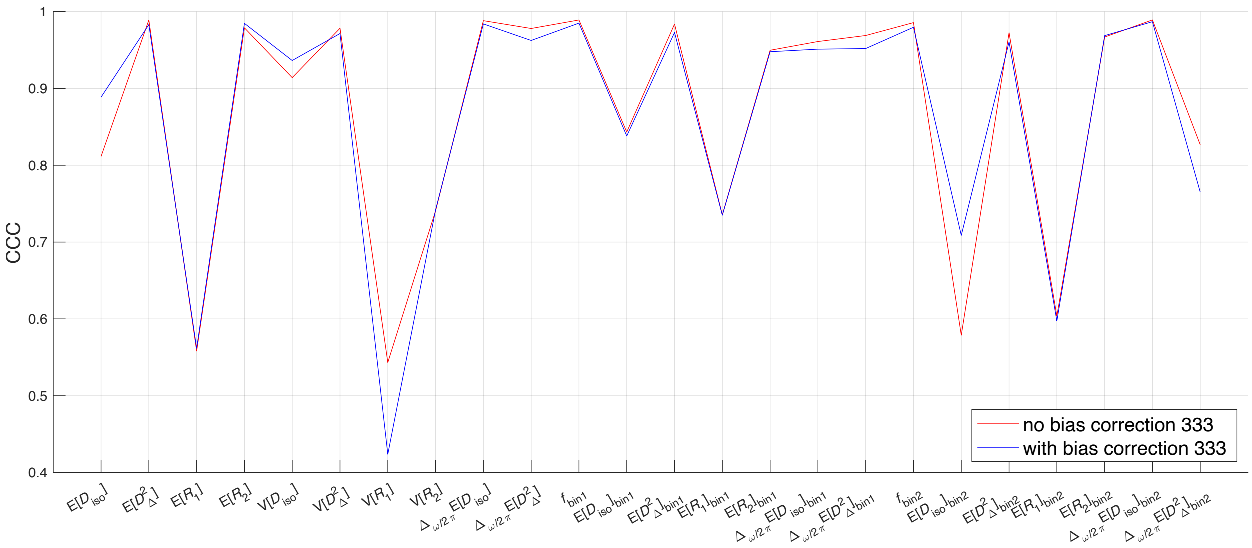

Supplementary Figure 3. CCC values computed from results with and without Rician bias correction, both denoised with MP-PCA patch size 3x3x3. Note the improvement in $E[D_{iso}]$ and $E[D_{iso}]_{bin2}$ when bias correction is applied. There is also simultaneously a decrease of a similar scale in CCC for $V[R_1]$.

References

1. Avants BB, Tustison NJ, Song G, Cook PA, Klein A, Gee JC. A reproducible evaluation of ANTs similarity metric performance in brain image registration. *NeuroImage*. 2011/02/01/ 2011;54(3):2033-2044. doi:10.1016/j.neuroimage.2010.09.025
2. 3D Slicer. Accessed Dec 29, 2024. https://www.slicer.org/
